# There Is No Association between MicroRNA Gene Polymorphisms and Risk of Triple Negative Breast Cancer in a Chinese Han Population

**DOI:** 10.1371/journal.pone.0060195

**Published:** 2013-03-26

**Authors:** Fei Ma, Ping Zhang, Dongxin Lin, Dianke Yu, Peng Yuan, Jiayu Wang, Yin Fan, Binghe Xu

**Affiliations:** 1 Department of Medical Oncology, Cancer Hospital, Chinese Academy of Medical Sciences and Peking Union Medical College, Beijing, China; 2 Department of Etiology and Carcinogenesis, Cancer Hospital, Chinese Academy of Medical Sciences and Peking Union Medical College, Beijing, China; The University of Texas M. D. Anderson Cancer Center, United States of America

## Abstract

Triple-negative breast cancer (TNBC) is defined by the lack of the expression of estrogen receptor (ER), progesterone receptor (PR) and human epidermal growth factor receptor 2 (HER2). It is characterized by aggressive behavior, poor prognosis and lack of targeted therapies. MicroRNA (miRNA) as a novel modulator of gene expression has played an important regulatory role in the malignancy. Dysregulation and/or mutation of the miRNAs may also contribute to the TNBC susceptibility since it is associated with the expression of ER, PR and HER2. Single nucleotide polymorphisms (SNPs) in miRNAs may be extremely relevant for TNBC. We tried to validate the hypothesis that genetic variations in miRNA are associated with TNBC development, and identify candidate biomarkers for TNBC susceptibility and clinical treatment. We screened the genetic variants in all miRNA genes listed in the public database miRBase and NCBI. A total of 23 common SNPs in 22 miRNAs, which tagged the known common variants in the Chinese Han people with a minor allele frequency greater than 0.05, were genotyped. This case-control study involved 191 patients with TNBC and 192 healthy female controls. Frequencies of SNPs were compared between cases and controls to identify the SNPs associated with TNBC susceptibility. No significant association was found between TNBC risk and the SNPs in the miRNA genes in the Chinese Han people (*P*>0.05), but this warrants further studies.

## Introduction

Triple-negative breast cancer (TNBC) is defined as a subgroup of breast carcinomas that are negative for expression of estrogen receptor (ER), progesterone receptor (PR) and human epidermal growth factor receptor-2(HER2) [1], [2]. TNBC accounts for approximately 10%–20.8% of all breast cancers, with a higher morbidity in younger women [3], [4], [5]. Furthermore, TNBC is characterized by aggressive behavior, poor prognosis and lack of targeted therapies [6], [7], [8]. It is reported that African American and Hispanic women have a higher risk of TNBC, and African Americans have worse prognosis than any other ethnic groups [5], [9], suggesting that genetic background may play an important role in TNBC and genetic variation would be a major risk for TNBC.

From the viewpoint of the human genome, individuals are 99.9% identical. Yet, the residual 0.1% leads to several million spelling differences, with some of the variations posing dramatically higher risks of certain cancers and other diseases. These differences are known as polymorphisms, of which the most important type is the single nucleotide polymorphisms (SNPs). SNP is a DNA sequence variation that occurs when a single nucleotide (A, T, C, or G) in the genome sequence is altered. Within a population, SNPs can be assigned a minor allele frequency, the ratio of chromosomes in the population carrying the less common variant to those with the more common variant. SNPs make up 90% of all human genetic variations, and a SNP with a minor allele frequency of ≥1% occurs every 1000 base pairs along the human genome. There are roughly 3×10^6^ SNPs in the human genome. In fact, the 0.1% of variations in the DNA sequences of humans can lead to various risks of diseases and affect how humans develop diseases and respond to pathogens, chemicals, drugs, etc. Consequently, SNPs as biological markers are of great value to biomedical research and can provide the information about a patient’s risk for disease development and the disease process, and protein targets for novel drug therapies. SNPs have been recently reported to be associated with the susceptibility and prognosis of breast cancer.

A microRNA (miRNA) is a short ribonucleic acid (RNA) molecule found in eukaryotic cells. A microRNA molecule has fewer nucleotides (an average of 22) compared with other RNAs, and there are 84% of miRNAs with a length range of 21–23 nucleotides [10]. miRNAs are post-transcriptional regulators that bind to complementary sequences on target messenger RNA transcripts (mRNAs), usually resulting in translational repression or target degradation and gene silencing. To date, there are a total of 695 human miRNAs available in the public database miRBase (http://microrna.sanger.ac.uk), and more than half of the miRNAs are located in the cancer-associated genomic regions or at fragile sites, as well as in the minimal regions of loss of heterozygosity, minimal regions of amplification (minimal amplicons), or common breakpoint regions [11]. MicroRNAs target about 60% of all genes, are abundantly present in all human cells and are able to regulate the expression of one-third of all human genes. The dysregulation of miRNA has been found to be closely associated with the development of various tumors [12]. Up to now, a few miRNAs acting as proto-oncogenes or tumor-suppressor genes, have been found to be involved in the development and progression of tumors by regulating the transcription and translation of the target genes [12], [13]. In breast cancer, the expression of ER, PR and HER2 is also regulated by miRNAs [14], [15].

Previous studies have focused on the regulations of miRNA on its target genes, but have neglected the abnormal expression and functions of miRNA itself. Most of the miRNA genes arise from discrete independent transcription units that are not located near to their target genes. The SNPs in miRNA genes, including pri-miRNAs, pre-miRNAs and mature miRNAs, could potentially influence the function of miRNA [16]. The genetic variations of miRNA-encoding genes can modify the function of miRNAs by changing the expression and maturation of miRNAs, including the selection of the target sites and the inhibitory effects on target genes of miRNAs [17]. The structure and function of miRNA genes are closely related to the development of tumors. In addition, some studies have reported that SNPs in miRNA genes are associated with the pathogenesis of multiple tumors [18], [19], [20], [21], [22]. In a case-control study of 346 Caucasian esophageal cancer patients and 346 frequency-matched (age, gender, and ethnicity) controls, seven SNPs in 26 miRNA-related genes were found to be significantly associated with esophageal cancer risk [19]. The SNPs in miRNA-related genes were also found to be closely associated with the risk of bladder carcinoma, renal carcinoma and non-small cell lung cancer [20], [21], [22].

Although several studies have focused on the association between individual SNPs of miRNA genes and breast cancer susceptibility [23], [24], there have been few systematical studies about the relationship between miRNA genetic variations and TNBC risk, especially in the Chinese women. Thus, in this case–control study, we detected the main SNPs of all miRNA genes in an attempt to discover the genetic variants in miRNA genes which alter the susceptibility to TNBC in a Chinese Han population.

## Materials and Methods

### Ethics Statement

This study was approved by the Institutional Review Board of the Chinese Academy of Medical Sciences Cancer Hospital (No: CH-BC-019).

### Study Subjects

All the subjects included in this case-control study were genetically independent ethnic Han Chinese. They consisted of 383 women who were permanent residents of Beijing and several other provinces in northern China. The subjects were divided into two arms. Arm 1 (n = 191) was TNBC patients, and arm 2 was the normal controls. All the eligible cases in arm 1 were confirmed histopathologically and treated at Cancer Hospital, Chinese Academy of Medical Sciences (Beijing, China). Patients with a previous history of cancer, metastatic cancer, or previous radiotherapy or chemotherapy were excluded. This study was approved by the Institutional Review Board of the Chinese Academy of Medical Sciences Cancer Hospital (No: CH-BC-019).

The ER and PR status was evaluated based on the immunohistochemical (IHC) results of formalin-fixed, paraffin-embedded breast cancer tissue samples from the patients. Positive ER and PR status was defined by nuclear staining of more than 10%. IHC was performed with anti-ER and anti-PR antibody. To determine the HER2 status, IHC or gene amplification was performed by fluorescence *in situ* hybridization (FISH). Tumors negative for ER, PR and HER2 were defined as TNBCs and compared with normal controls.

The control series consisted of 192 unrelated female blood donors, who were randomly drawn from the Breast Cancer Screening Project of the same hospital during the same period, and presented no evidence of breast cancer, or any other suspicious precancerous lesions of the breast. These normal controls reported no cancer history and were frequency-matched to the cases on age (±5 years).

### Candidate miRNAs and SNP Selection

We screened genetic variants in all miRNA genes, which are listed in the public database miRBase (http://microrna.sanger.ac.uk) and NCBI (http://www.ncbi.nlm.nih.gov/SNP/). The common SNPs in pre-miRNA and flanking sequences (mainly in the 2 kb upstream regulatory region) were selected as candidates for genotyping. Although there are a large number of human miRNAs, their encoding sequences are short and highly conserved. Therefore, the common SNPs in human miRNAs are limited in number. The inclusion criteria for candidate SNPs were as follows: SNPs known in ethnic Han Chinese people; SNPs with a minor allele frequency (MAF) of >0.05. Finally, a total of 24 candidate SNPs were selected for genotyping.

### Genomic DNA Extraction

Genomic DNA was extracted by Phenol/Chloroform. Two ml blood sample was collected from the patients and controls, and stored at −80°C. The frozen samples were thawn and centrifuged at 5000×*g* for 15 min, and the upper layer was removed and discarded. An appropriate amount of lysis buffer (10 mM Tris-HCl, pH 8.0; 0.1 M EDTA; 20 µg/ml RNase A, 0.5% SDS) was added into the tube and mixed with the cell pellet thoroughly. The mixture was incubated at 37°C for 1 hour. Proteinase K solution (100 µg/ml) was added and mixed thoroughly, and incubated at 37°C overnight. An equal volume of Tris-HCl buffer-saturated phenol solution (pH 7.4) was then added into the mixture, mixed thoroughly and centrifuged at 8000×*g* for 15 min. The upper aqueous layer was carefully removed to a new tube and an equal volume of phenol:chloroform (1∶1) was added to the solution, mixed thoroughly and centrifuged at 8000×*g* for 15 min. The upper aqueous layer was removed to a new tube, added with 10% ammonium acetate solution (10 M), mixed thoroughly, added with 2 volumes of ethanol, mixed well and stored at −20°C. The DNA precipitation was washed twice by 75% ethanol and then dissolved in TE buffer. The DNA concentration was determined by a spectrophotometer. The extracted DNA sample was placed into a 1.5 ml micro-centrifugal tube and stored at −80°C. The requirements for Sequenom analysis are: DNA concentration ≥10 ng/µl, and OD260/280 = 1.8–2.0.

### Genotyping and Quality Control

MassARRAY® MALDI-TOF System (Sequenom Inc., San Diego, CA, USA) was used for genotyping the candidate SNPs by the method described in Sequenom Genotyping Protocol. The PCR primers and probes were designed according to the reference sequences in NCBI GenBank database. Table 1 displays the PCR primers and probes of the detected 24 SNPs. Duplicate samples and negative controls (without DNA) were set for quality assurance of genotyping. Concordance for duplicate samples was 100% for all assays. The group information of each sample was blinded to genotyping analysis and the analysts.

**Table 1:** PCR primers and probes of candidate SNPs of miRNAs.

SNP		PCR primers	Extend primer
Has-miR-10b	rs1867863	forward primer:ACGTTGGATGTCAGAAAGTGCGGGTGGCTG	CTTCCTCTGAGCCAC
		reverse primer:ACGTTGGATGTGAGGTACCTAGGTCGCTG	
hsa-mir-27a	rs895819	forward primer:ACGTTGGATGAGCAGGGCTTAGCTGCTTGT	TGTGAACACGACTTGG
		reverse primer:ACGTTGGATGACTTAGCCACTGTGAACACG	
hsa-mir-423	rs6505162	forward primer:ACGTTGGATGTTTTCCAAAAGCTCGGTCTG	gggatAAACTCAAGCGCGGG
		reverse primer:ACGTTGGATGCAAGCGGGGAGAAACTCAAG	
hsa-mir-492	rs2289030	forward primer:ACGTTGGATGAGCACCCAAACACTCAAACC	ACTTGGTTCAGAAGTCAT
		reverse primer:ACGTTGGATGGGATTGTCCTGCAGATCAAC	
hsa-mir-603	rs11014002	forward primer:ACGTTGGATGATTGCAGTGTGTGCCATTAC	cTGGTGCAAAAGTAATTGCAGTG
		reverse primer:ACGTTGGATGGGCAAGATTGATGCTGTTGG	
hsa-mir-604	rs2368392	forward primer:ACGTTGGATGAAAACCTGCCTGCTAGTGGA	GTGCTTGACCTTCCA
		reverse primer:ACGTTGGATGCATGCACTGAGAGAGGTGTC	
hsa-mir-605	rs2043556	forward primer:ACGTTGGATGTGTGGCTGTCAGCCTGTAAC	aagatAAGGCACTATGAGATTTAGAA
		reverse primer:ACGTTGGATGAACAGAGAAGGCACTATGAG	
hsa-mir-608	rs4919510	forward primer:ACGTTGGATGAAGATCCACTGGGCCAAGGT	GGAGATGCCTTTTTAAACG
		reverse primer:ACGTTGGATGATGGAAGCTCTTGGAGATGC	
hsa-mir-100	rs1834306	forward primer:ACGTTGGATGGAAAAAGTGGAAACCAAGGG	tttgTCTTCTATGTTCTCCCCA
		reverse primer:ACGTTGGATGGTCCCACTCTCACAAAAGC	
hsa-mir-105-1	rs5970293	forward primer:ACGTTGGATGCTGAGCAGTTTTTACTCTGTG	GGATAGATATGGTATCCCAG
		reverse primer:ACGTTGGATGGGAGAATAGGTCAATTCATAC	
hsa-mir-105-2	rs5970292	forward primer:ACGTTGGATGAGAAGGAGGCAAACACACTG	TCTGTACCATGAATATAATTTTGTG
		reverse primer:ACGTTGGATGAAAATGGAATTTTCTGTACC	
hsa-mir-1206	rs2114358	forward primer:ACGTTGGATGCATGTAGATGTTTAAGCTC	tccGCGTTCACTAGCTTGCAAAAA
		reverse primer:ACGTTGGATGGTGTGGCGAGCATAATTTGG	
hsa-mir-1274a	rs318039	forward primer:ACGTTGGATGAGTAACTGTCGTAGTGATGG	ccCCCTGTTCAGGCGCCAC
		reverse primer:ACGTTGGATGTCCCTGTTTGTCCCTGTTC	
hsa-mir-125b-1	rs2081443	forward primer:ACGTTGGATGCACACATACGCAGTATCTGG	ctagCGTATGTGTATGTGCGTGATTGT
		reverse primer:ACGTTGGATGCTCGTGATCGTATGTGTATG	
hsa-mir-146a	rs2910164	forward primer:ACGTTGGATGCCACGATGACAGAGATATCC	ttggTGTGTCAGTGTCAGACCT
		reverse primer:ACGTTGGATGGAACTGAATTCCATGGGTTG	
hsa-mir-373	rs12983273	forward primer:ACGTTGGATGTAAACTTGCTTGCTATGGG	gGTTGGTGTTATAATTGATATGTAA
		reverse primer:ACGTTGGATGGGAATGCTTTTTGCTGTTGG	
hsa-mir-26a-1	rs7372209	forward primer:ACGTTGGATGCAGTCATGCTTACAGTCACG	AATTAGGAGAGAAATTAATCCTT
		reverse primer:ACGTTGGATGAAAGGAGAGGCTGCCCAATG	
hsa-mir-124a-1	rs531564	forward primer:ACGTTGGATGACAGACAGGGGCTTAGAGA	ggggtTTTCTCTCCCTGAGTCT
		reverse primer:ACGTTGGATGTGCACAGATCTGCTTCTGTC	
hsa-let-7f-2	rs17276588	forward primer:ACGTTGGATGGTACACTGCAAAAAGAAGGG	TAGGTGCTTGCAAAGT
		reverse primer:ACGTTGGATGGGTGACTATTACAGTTGACC	
hsa-mir-149	rs2292832	forward primer:ACGTTGGATGTCTTCACTCCCGTGCTTGTC	GACCTGCGTTGTTCC
		reverse primer:ACGTTGGATGAACTCGCCCAGCCGGCCC	
hsa-mir-196a2	rs11614913	forward primer:ACGTTGGATGTCGACGAAAACCGACTGATG	aaTCGGCAACAAGAAACTG
		reverse primer:ACGTTGGATGCTGATCTGTGGCTTAGGTAG	
hsa-mir-30c-1	rs16827546	forward primer:ACGTTGGATGCCTGACCACTGACACAGAAC	ggagGTGTCACACAGGTCA
		reverse primer:ACGTTGGATGGTTTTTGTTGTCCCAGGCAG	
hsa-mir-373	rs10425222	forward primer:ACGTTGGATGAAGCTTTTCCTGCCCTGTTC	gggtCAGGATACGGGGTTCA
		reverse primer:ACGTTGGATGCACCAGTCTGTATGCTCCAT	
hsa-mir-943	rs1077020	forward primer:ACGTTGGATGTAGCCTAGAAGAGGGTGGGA	CCCCACCCCCTGAGC
		reverse primer:ACGTTGGATGTGGAGGACGGCAACAGTCAG	

### Statistical Analysis

Hardy-Weinberg equilibrium test was undertaken to validate the genotype distributions of each SNP using the Chi-squared test or Fisher’s exact test (when sample size was too small for the χ^2^ test, the Fisher’s exact test was used). The SNPs whose distribution frequencies were not consistent with Hardy-Weinberg equilibrium would be kicked out of the association analysis. The χ^2^ test was used to examine differences in alleles and distribution of genotypes between cases and controls. The association between genotype and risk of breast cancer was estimated by calculating the odds ratio (OR) and their 95% confidence interval (95%CI) with unconditional logistic regression models. The ORs were adjusted for confounding factors such as age and tobacco smoking. All statistical tests were two-sided, and *P*<0.05 was considered significant. The statistical analyses were performed using SPSS10.0 and SHEsis [25] Statistical Analysis System software.

## Results

### Subject Characteristics

This study included 191 TNBC patients and 192 control subjects, the median age was 49 years (range, 21–81 years). The characteristics of the subjects are summarized in Table 2. There were no significant differences in age distribution (*P* = 0.161) and smoking status (*P* = 0.503) between patients and controls (*P* = 0.161). However, much more patients had a family history of breast cancer or ovarian cancer than the controls (10.5% *vs*. 1.6%, *P* = 0.000). In addition, there were more patients with menarche age below 14 years compared with the controls (48.7% *vs.* 37.5%, *P* = 0.030). And the number of premenopausal patients was also larger than that of the controls (62.3% *vs.* 50.0%, *P* = 0.018). All these characteristics were considered as common risks for breast cancer.

**Table 2:** Baseline clinical characteristics of TNBC patients and controls.

	Cases (n = 191)	Controls (n = 192)	*P* value^a^
	No.(%)	No.(%)	
Age(years)			0.161
≤40	43 (22.5)	56 (29.2)	
>40	148 (77.5)	136 (70.8)	
Family history of breast cancer or ovarian cancer^b^			0.000
No	171 (89.5)	189 (98.4)	
Yes	20 (10.5)	3 (1.6)	
Age of menarche (years)			0.030
≤14	93 (48.7)	72 (37.5)	
>14	98 (51.3)	120 (62.5)	
Menopausal status at diagnosis			0.018
Premenopausal	119 (62.3)	96 (50.0)	
Postmenopausal	72 (37.7)	96 (50.0)	
Smoking level (life-time cigarettes smoked)			0.503
<20	187 (97.9)	186 (96.9)	
≥20	3 (1.6)	6 (3.1)	
Unknown	1 (0.5)	0 (0)	

a Two-sided χ2 test.

b First-degree relative or second-degree relative with breast cancer or ovarian cancer.

### Genotyping and Association Analysis of miRNA SNPs and TNBC Risk

We screened genetic variants in all the miRNA genes listed in the public database miRBase and NCBI. A total of 24 common SNPs in 23 miRNAs were selected for genotyping (Table 3). The SNP rs11014002 was eliminated in the statistical analysis because it was not in accordance with the Hardy-Weinberg equilibrium. Finally, a total of 23 SNPs in 22 miRNAs were included in the statistical analysis. Table 4 shows the genotype frequencies of all miRNA SNPs in both cases and controls. The non-conditional logistic regression analysis found no statistically significant differences between cases and controls in terms of distribution frequencies of SNP genotypes. Moreover, the SNP genotypes of patients and controls were not obviously associated with TNBC risk (*P*>0.05).

**Table 3:** A list of single nucleotide polymorphisms in candidate miRNA genes.

miRNA name	Localization	Entrez SNP ID^a^	Alleles (major/minor)	MAF%	HWE^b^ *P*	Targets
hsa-mir-146a	5q34	rs2910164	C/G	42.1	1.0000	BRCA1/2
hsa-mir-10b	2q31.1	rs1867863	A/C	35.9	0.4064	E2F7;MAP3K2
hsa-mir-373	19q13.42	rs12983273	C/T	6.8	0.3866	NF1B;MAP3K14
	19q13.42	rs10425222	C/A	9.3	1.0000	LATS2
hsa-mir-27a	19p13.12	rs895819	T/C	25.8	0.7060	Sp;MAP3K4
hsa-mir-423	17q11.2	rs6505162	C/A	23.5	1.0000	NFKBIL2
hsa-mir-492	12q22	rs2289030	C/G	23.0	0.8379	CDK9;MAP3K14
hsa-mir-124-1	8p23.1	rs531564	C/G	16.1	0.1618	MAPK14
hsa-mir-603	10p12.2	rs11014002	C/T	7.0	5.97×10^−8^	NF1B
hsa-mir-604	10p11.23	rs2368392	C/T	27.9	1.0000	NFRKB
hsa-mir-26a-1	3p22.2	rs7372209	C/T	28.8	0.5283	PTEN
hsa-mir-605	10q21.1	rs2043556	A/G	30.0	0.0034	TRPM4
hsa-mir-608	10q24.31	rs4919510	G/C	46.3	0.0863	MAP2K2
hsa-mir-100	11q24.1	rs1834306	C/T	48.2	0.3002	IGF1R
hsa-mir-105-1	Xq28	rs5970293	G/C	27.5	0.3997	BRAP
hsa-mir-105-2	Xq28	rs5970292	G/A	23.7	1.0000	KRAS;CDK9
hsa-mir-1206	8q24.21	rs2114358	T/C	22.8	0.0208	MAPK14;KRAS
hsa-mir-1274a	5p13.1	rs318039	C/T	13.8	0.5215	E2F2;NF1B
hsa-mir-125b-1	11q24.1	rs2081443	T/G	33.7	0.8453	APC
hsa-mir-943	4p16.3	rs1077020	C/T	24.3	0.0062	BMP3;GAS2
hsa-mir-196a-2	12q13.13	rs11614913	T/C	47.3	0.0474	p27Kip1;E2F7
hsa-mir-30c-1	1p34.2	rs16827546	C/T	4.5	0.6301	CASP3;PTEN
hsa-let-7f-2	Xp11.22	rs17276588	G/A	28.7	0.6900	IGF1R;MAPK6;RAS CASP3
hsa-mir-149	2q37.3	rs2292832	T/C	30.1	0.0026	CCN1;TOP1

a Entrez SNP reference ID(http://www.ncbi.nlm.nih.gov/entrez/query.fcgi?db=np).

b Hardy-Weinberg equilibrium test was undertaken to calculate the *P* value, and *P*<0.001 was considered not in accordance with Hardy-Weinberg equilibrium.

**Table 4:** Association between genotypes and TNBC risk.

SNP ID (miRNA name)	Genotypes	Distribution frequencies of genotypes		OR(95%CI)^a^	*P* value
		Case	Control		
rs12983273 (hsa-mir-373)	CC	161(0.8610)	160(0.8743)	0.8901[0.4874,1.6255]	0.7258
	TT	1(0.0053)	2(0.0109)	0.4866[0.0437,5.4130]	
	CT	25(0.1337)	21(0.1148)	1.1905[0.6406,2.2123]	
rs10425222 (hsa-mir-373)	CC	149(0.7968)	154(0.8191)	0.8657[0.5175,1.4483]	0.8571
	AA	1(0.0053)	1(0.0053)	1.0054[0.0624,16.1940]	
	AC	37(0.1979)	33(0.1755)	1.1586 [0.6886,1.9492]	
rs1834306 (hsa-mir-100)	CC	60(0.3141)	55(0.2895)	1.1242[0.7256,1.7417]	0.4544
	TT	38(0.1990)	48(0.2526)	0.7347[0.4533,1.1910]	
	CT	93(0.4869)	87(0.4579)	1.1235[0.7513,1.6802]	
rs2043556 (hsa-mir-605)	AA	42(0.4330)	68(0.4387)	0.9770[0.5856,1.6300]	0.9924
	GG	4(0.0412)	6(0.0387)	1.0681[0.2936,3.8857]	
	AG	51(0.5258)	81(0.5226)	1.0129[0.6094,1.6836]	
rs2081443 (hsa-mir-125b-1)	TT	98(0.5185)	81(0.4332)	1.4093[0.9387,2.1157]	0.2501
	GG	18(0.0952)	20(0.1070)	0.8789[0.4491,1.7203]	
	GT	73(0.3862)	86(0.4599)	0.7391[0.4903,1.1141]	
rs2114358 (hsa-mir-1206)	TT	110(0.5729)	108(0.5654)	1.0309[0.6880,1.5449]	0.2333
	CC	10(0.0521)	4(0.0209)	2.5687[0.7914,8.3375]	
	CT	72(0.3750)	79(0.4136)	0.8506[0.5644,1.2821]	
rs2289030 (hsa-mir-492)	CC	127(0.6615)	114(0.5969)	1.3197[0.8707,2.0002]	0.1986
	GG	5(0.0260)	11(0.0576)	0.4375[0.1491,1.2842]	
	CG	60(0.3125)	66(0.3455)	0.8609[0.5619,1.3190]	
rs2368392 (hsa-mir-604)	CC	99(0.5156)	99(0.5211)	0.9785[0.6550,1.4618]	0.9923
	TT	15(0.0781)	15(0.0789)	0.9887[0.4691,2.0838]	
	CT	78(0.4063)	76(0.4000)	1.0263[0.6819,1.5448]	
rs2910164 (hsa-mir-146a)	CC	63(0.3281)	64(0.3351)	0.9691[0.6333,1.4831]	0.9875
	GG	35(0.1823)	34(0.1780)	1.0294[0.6113,1.7336]	
	CG	94(0.4896)	93(0.4869)	1.0108[0.6770,1.5090]	
rs318039 (hsa-mir-1274a)	CC	146(0.7604)	142(0.7513)	1.0505[0.6582,1.6767]	0.762
	TT	3(0.0156)	5(0.0265)	0.5841[0.1376,2.4795]	
	CT	43(0.2240)	42(0.2222)	1.0101[0.6235,1.6363]	
rs4919510 (hsa-mir-608)	GG	57(0.2969)	61(0.3211)	0.8929[0.5783,1.3785]	0.2544
	CC	37(0.1927)	47(0.2474)	0.7263[0.4463,1.1820]	
	CG	98(0.5104)	82(0.4316)	1.3731[0.9176,2.0548]	
rs531564 (hsa-mir-124-1)	CC	126(0.6923)	136(0.7196)	0.8768[0.5608,1.3711]	0.3519
	GG	4(0.0220)	8(0.0423)	0.5084[0.1504,1.7186]	
	CG	52(0.2857)	45(0.2381)	1.2800[0.8047,2.0361]	
rs5970292 (hsa-mir-105-2)	GG	93(0.5254)	111(0.5842)	0.7845[0.5217,1.1798]	0.5057
	AA	22(0.1243)	11(0.0579)	1.1287[0.4851,2.6262]	
	AG	62(0.3503)	68(0.3579)	1.2509[0.8245,1.8979]	
rs5970293 (hsa-mir-105-1)	GG	119(0.6230)	102(0.5397)	1.1099[0.7406,1.6633]	0.3608
	CC	12(0.0628)	17(0.0899)	0.5590[0.2490,1.2547]	
	CG	60(0.3141)	70(0.3704)	1.0517[0.6944,1.5929]	
rs6505162 (hsa-mir-423)	CC	127(0.6615)	110(0.5820)	1.4032[0.9258,2.1267]	0.278
	AA	8(0.0417)	10(0.0529)	0.7783[0.3003,2.0168]	
	AC	57(0.2969)	69(0.3651)	0.7343[0.4784,1.1270]	
rs7372209 (hsa-mir-26a-1)	CC	109(0.5677)	99(0.5183)	1.2204[0.8159,1.8254]	0.5407
	TT	19(0.0990)	18(0.0942)	1.0556[0.5357,2.0799]	
	CT	64(0.3333)	74(0.3874)	0.7905[0.5204,1.2009]	
rs895819 (hsa-mir-27a)	TT	97(0.5132)	106(0.5579)	0.8355[0.5577,1.2516]	0.6783
	CC	16(0.0847)	14(0.0737)	1.1627[0.5507,2.4549]	
	CT	76(0.4021)	70(0.3684)	1.1530[0.7621,1.7444]	
rs1867863 (hsa-mir-10b)	AA	78(0.4105)	74(0.3936)	1.0729 [0.7111,1.6187]	0.8465
	CC	30(0.1579)	21(0.1117)	1.4911 [0.8197,2.7124]	
	AC	82(0.4316)	93(0.4947)	0.7756 [0.5172,1.1630]	
rs1077020 (hsa-mir-943)	CC	105(0.5707)	100(0.5348)	1.1563 [0.7677,1.7417]	0.696
	TT	5(0.0272)	4(0.0214)	1.2779 [0.3377,4.8361]	
	CT	74(0.4022)	83(0.4439)	0.8429 [0.5581,1.2732]	
11614913 (hsa-mir-196a2)	TT	54(0.2842)	59(0.3155)	0.8614 [0.5542,1.3389]	0.4833
	CC	44(0.2316)	49(0.2620)	0.8488 [0.5311,1.3565]	
	CT	92(0.4842)	79(0.4225)	1.2834 [0.8548,1.9269]	
rs16827546 (hsa-mir-30c-1)	CC	165(0.8730)	172(0.9149)	0.6395 [0.3280,1.2469]	0.4066
	TT	2(0.0106)	1(0.0053)	2.0000 [0.1798,22.2466]	
	CT	22(0.1164)	15(0.0798)	1.5194 [0.7622,3.0288]	
rs17276588 (hsa-let-7f-2)	GG	92(0.4894)	97(0.5160)	0.8991 [0.6000,1.3472]	0.8754
	AA	18(0.0957)	17(0.0904)	1.0651 [0.5310,2.1363]	
	AG	78(0.4149)	74(0.3936)	1.0924 [0.7235,1.6494]	
rs2292832 (hsa-mir-149)	TT	99(0.5351)	100(0.5376)	0.9900 [0.6583,1.4889]	0.2846
	CC	17(0.0919)	26(0.1398)	0.6227 [0.3256,1.1911]	
	CT	69(0.3730)	60(0.3226)	1.2491 [0.8141,1.9167]	

a Adjusted for age and smoking status.

*P* trend : calculated by Cochran–Armitage trend test.

## Discussion

Triple-negative breast cancer (TNBC) is defined as a subgroup of breast carcinomas. TNBC exhibits special biological and clinicopathological characteristics, and have high proliferation and low differentiation ratios. TNBC shares some similar characteristics with basal-type breast cancers and BRCA1-related breast cancers. TNBCs are generally very sensitive to chemotherapy; however, some types of TNBCs are known to be more aggressive with poor prognosis. TNBC arises as a result of multiple somatic molecular events that can be genetic or epigenetic. Genetic variability appears to influence not only the risk but also the type of TNBC. Therefore, it is necessary to investigate the pathogenesis of TNBC and search for the genetic markers which can predict the development of TNBC.

MicroRNAs (miRNA) are small non-coding RNA molecules involved in a diversity of cellular functions. miRNA, as a “hacker” in gene research, can regulate the expression of one-third of all human genes. A number of studies have shown that dysregulation of miRNAs is involved in cancer initiation and progression. However, few studies have investigated the association between genetic variants in miRNA genes and TNBC susceptibility. We thus attempted to investigate the SNPs of all miRNA genes in an ethnic Han Chinese population using the MassARRAY® MALDI-TOF System. We hypothesized that genetic variations of the miRNA genes could be associated with the risk of TNBC. However, the results showed that none of the SNPs was significantly associated with TNBC. This finding may be attributed to a number of reasons as follows.

Firstly, the majority of miRNA genes are highly conserved [26], especially the seed sequences which bind to the target messenger RNA transcripts (mRNAs). Some recent studies have found SNPs in miRNA genes [16]. Iwai et al. [27] sequenced 173 human pre-miRNA genome regions in 96 subjects and identified 10 polymorphisms in 10 pre-miRNA hairpin regions. They identified a C to A polymorphism in the mature miR-30c-2 sequence which may alter target selection, thus exerting profound biological effects; however, the other 9 polymorphisms have shown no effect on microRNA processing. These results are consistent with the conservative property of miRNA genes. We found no genetic variants in miRNA genes which are associated with the development of TNBC in this study, possibly because SNPs in miRNA genes can not alter target selection of miRNAs, thus can not exert any biological effects.

Secondly, miRNA-related SNP is a wide concept, which involves SNPs in miRNA encoding genes, regulatory factors of miRNA transfer, processing and maturation pathways and SNPs in miRNA target site. These SNPs can alter the function of miRNA by different ways and mechanisms. We have investigated the SNPs in miRNA encoding genes; however, it is just the tip of the iceberg of miRNA’s complex regulatory network. Although SNPs in miRNA encoding genes were not found associated with TNBC risk in the ethnic Han Chinese population, these negative results seem to suggest that SNPs in miRNA target site or in regulatory factors of miRNA, compared with SNPs in miRNA encoding genes, may be more target and/or pathway specific. Some studies have supported this viewpoint. Nicoloso et al [28] analyzed target SNPs, which were known to modify miRNA binding sites and miRNA gene regulation, and found that target SNPs were implicated in breast caner susceptibility, and germline occurrence of rs799917-BRCA1 and rs334348-TGFR1 significantly varied among populations at different risks for breast caner development. Liang et al [29] have analyzed 226 SNPs in miRNA processing genes and miRNA binding sites in 339 ovarian cancer cases and 349 healthy controls, and found that 13 SNPs were significantly associated with ovarian cancer risk. In addition, Saunders et al [16] analyzed the publicly available SNP data in context with miRNAs and their target sites throughout the human genome, and found a relatively low level of variation in functional regions of miRNAs, but an appreciable level of variation at target sites.

Thirdly, miRNA plays a very complex role in the regulation of tumors. Compelling evidence has shown that miRNAs are involved in cancer initiation and progression by gene amplification [30], [31], [32], [33], gene deletion [34], and abnormal activation or inhibition of proteins which can regulate the expression of miRNAs [13], [35], [36], [37]. On the other hand, SNPs in miRNA genes, including pri-miRNAs, pre-miRNAs and mature miRNAs, could potentially influence the function of miRNA. The genetic variations of miRNA-encoding genes can modify the function of miRNAs by changing the expression and maturation of miRNAs, including the selection of the target sites and the inhibitory effects on target genes of miRNAs. Over the past few years, an increasing number of studies have highlighted the key role of the microRNAs in the regulation of several important signal pathways of tumorigenesis and apoptosis. In this study, we have screened SNPs, whose minor allele frequency (MAF) is listed in the public database miRNA Base and NCBI, in all miRNA genes of ethnic Han Chinese population. And we selected SNPs located in various miRNAs, including miRNAs which regulate breast cancer-associated genes, such as RAS, PTEN, ATM and BRCA1/2 (hsa-mir-149, hsa-mir-196a-2, hsa-mir-30c-1, hsa-mir-146a, hsa-let-7f-2); miRNAs regulating breast cancer associated receptors ER, PR and HER2 (hsa-mir-27a, hsa-mir-125b-1, hsa-mir-105-1, hsa-mir-105-2); and miRNAs closely associated with breast caner invasion and metastasis (hsa-mir-373 and hsa-mir-10b). The synergic effect of the miRNA genes plays a very important role in the generation, extinction and evolution of miRNA itself [38]. The complex regulatory network between miRNAs and various factors which could influence the expression of miRNAs, can both mask the biological effects of a single SNP. Therefore, we observed no significant association between SNPs in miRNA genes and TNBC risk. We will determine whether polymorphic sites in miRNA genes with linkage relationship will alter the association analysis between SNPs and disease susceptibility in our upcoming studies.

Finally, this is a preliminary exploratory study. When designing this study, we focused on the main effects of predisposing genes, but not on the gene-gene interactions. The sample size was small (cases/controls, 191/192), which may lead to the negative result and its consequent low power of test. This is mainly attributed to the fact that the incidence of TNBC is low, being about 12% of all the breast cancers in Chinese people.

In conclusion, this is the first study to investigate the relationship between all miRNAs with genetic variants and TNBC risk in a Chinese Han population, which has shown that SNPs in all miRNAs were not obviously associated with TNBC risk. MicroRNAs play an important role in physiological and pathological processes. They interact with each other intricately and exert complex functions which have not been clearly elucidated. In addition, miRNAs are highly conserved. The genotype and function of SNPs in miRNA-encoding genes and their influence on phenotypes are also unknown. In the present study, we have found no significant association between the miRNA gene polymorphisms and TNBC risk in ethnic Han Chinese population. These findings suggest that genetic polymorphisms in miRNA-encoding genes, due to its inherent characteristics, may have little contribution to the research of population genetics. However, further investigation is needed to validate these results.
